# Characterization of aerosols containing *Legionella* generated upon nebulization

**DOI:** 10.1038/srep33998

**Published:** 2016-09-27

**Authors:** Séverine Allegra, Lara Leclerc, Pierre André Massard, Françoise Girardot, Serge Riffard, Jérémie Pourchez

**Affiliations:** 1 University of Lyon, UJM-Saint-Etienne, CNRS, EVS-ISTHME UMR 5600, F42023 Saint-Etienne, France; 2 University of Lyon, UJM-Saint-Etienne, GIMAP, EA 3064, F-42023, F-42023 Saint-Etienne, France; 3 University of Lyon, Ecole Nationale Supérieure des Mines-Saint-Etienne, CIS-EMSE, SAINBIOSE, INSERM U1059, F-42023 Saint Etienne, France

## Abstract

*Legionella pneumophila* is, by far, the species most frequently associated with Legionnaires’ disease (LD). Human infection occurs almost exclusively by aerosol inhalation which places the bacteria in juxtaposition with alveolar macrophages. LD risk management is based on controlling water quality by applying standardized procedures. However, to gain a better understanding of the real risk of exposure, there is a need (i) to investigate under which conditions *Legionella* may be aerosolized and (ii) to quantify bacterial deposition into the respiratory tract upon nebulization. In this study, we used an original experimental set-up that enables the generation of aerosol particles containing *L. pneumophila* under various conditions. Using flow cytometry in combination with qPCR and culture, we determined (i) the size of the aerosols and (ii) the concentration of viable *Legionella* forms that may reach the thoracic region. We determined that the 0.26–2.5 μm aerosol size range represents 7% of initial bacterial suspension. Among the viable forms, 0.7% of initial viable bacterial suspension may reach the pulmonary alveoli. In conclusion, these deposition profiles can be used to standardize the size of inoculum injected in any type of respiratory tract model to obtain new insights into the dose response for LD.

*Legionella* are facultative intracellular pathogens that cause Legionnaires’ disease (LD), although some species are strictly intracellular^1^. Epidemiological studies unambiguously identified water as the main reservoir of *Legionella*. Man-made ecosystems (e.g., hot water and air conditioning systems, shower heads, humidifiers, cooling towers, and whirlpool baths) have been the source of several outbreaks of LD^2^,^3^,^4^,^5^. In particular, hospital water distribution systems serve as the primary reservoir for nosocomial legionellosis. Respiratory therapeutic devices (including oxygen humidifiers, medical aerosol-producing devices such as nebulizers and face masks) filled or rinsed with tap water may serve as secondary reservoirs for LD infection^6^,^7^,^8^. LD may also occur after inhalation of amoeba vacuoles containing *Legionella*^9^.

A bioaerosol is defined as a collection of biological particles (including bacteria and fungi in addition to viruses and fragments of organisms) dispersed in the air^10^. It is generally composed of single cells or aggregates of cells (e.g., bacterial cells, spores, actinomycetes, endotoxins, and amoebae vacuoles) but can also be composed of cell conglomerations with small dust particles or water droplets. For instance, LD is a high-profile pathogenic bioaerosol-transmitted infection^11^,^12^,^13^.

The ability of *Legionella* to cause disease is associated with their ability to enter the respiratory system through respirable-size droplets as transporting particles. The conditions under which *Legionella* may be aerosolized and how they enter the respiratory tract upon aerosol generation remain poorly documented^10^,^14^.

Droplet generation is thought to be a key step in the transmission of *Legionella*^4^; water is as an excellent transporting medium for the bacterium. However, the effectiveness of the process by which water in the reservoir is transferred to the air and then to the human respiratory tract remains poorly investigated. Both processes seem to depend mainly on the interdependence of the sizes of transporting and transported particles^15^. The geometric and aerodynamic sizes of the water droplets with respect to microbial survival and lung deposition are critical factors in the delivery of viable bacteria to the human respiratory system. In addition, the process at the origin of water droplet generation can induce enrichment of bacterial concentration in the airborne liquid droplets relative to the bulk concentration in the reservoir^15^. This bacterial enrichment is a complex phenomenon. As a result, measurement of the size distribution of liquid aerosols correlated with an infectious dose remains challenging. Infectious doses for humans can be extrapolated only from animal inhalation studies (e.g., guinea pigs) in the CFU range of 8,000–17,000^16^. Of the mechanisms by which human infection may occur, we decided to focus on medical nebulization because it has been less studied in comparison with other environmental exposures, such as aerosols generated from spa pools or industrial cooling towers^4^,^17^. Our study benefits from the newest technology available on the nebulizer market for droplet generation, vibrating-mesh nebulizers, rather than the common jet nebulizers commonly used to infect animals during inhalation experiments^14^. The main advantages of vibrating-mesh nebulizers include consistent aerosol generation efficiency, a high fine-particle fraction reaching the peripheral lung and a low residual volume in the reservoir. It is commonly accepted that mesh nebulizers are more efficient than jet nebulizers and can provide higher drug doses to patients^18^.

In addition to investigating the effectiveness of the *L. pneumophila* transfer from the water in the reservoir to the air and then to the human respiratory tract, we evaluated the viability of the inoculum within the generated droplets. At least 14 physiological forms of *L. pneumophila* have been described according to the various environments they may colonize^19^. Among them, the presence in water samples of viable but not culturable (VBNC) forms contributes to underestimation of the number of potentially pathogenic *Legionella* detected by standard methods. Our preliminary studies on anthropic reservoirs have demonstrated the presence of VBNC forms of *Legionella* after water disinfection treatments (heat^20^ and chlorine^21^). Finally, the role of *Legionella* intracellular forms (intra-amoebic) has been little investigated in environmental samples^22^ even though these forms are considered more infectious^19^. VBNC cells can be detected and quantified by a flow cytometric assay (FCA)^23^. Assessment of their presence is important because they are able to infect amoebas as well as macrophage-like and epithelial cells from human respiratory mucosa after resuscitation on amoebas^21^,^24^.

Very few studies have sought to quantify the presence of all viable forms of *Legionella* and size of *Legionella*-containing droplets^25^,^26^. Thus, in this paper, we propose a methodology for determining airborne particle distribution and the number of *Legionella* cells in airborne droplets of a given size. More specifically, new insights are provided into the size characterization of *Legionella* bioaerosols, including assessment of the number of viable (culturable (VC) or not (VBNC)) bacterial cells in respirable droplet size fractions. The secondary objectives were (i) to improve our knowledge of the role of aerosols in the transmission of *Legionella* using vibrating-mesh nebulization and (ii) to fully characterize an experimental set-up that enables determination of aerosol deposition profiles under laboratory conditions within an animal respiratory tract.

## Results and Discussion

### Validation of the experimental set-up

**Figure 1:**
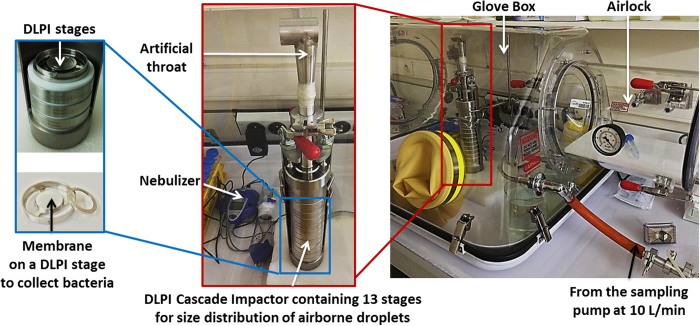
Experimental set-up for the generation and characterization of aerosols.

Aerosol generation by an e-Base^®^ vibrating-mesh nebulizer was performed in a glove box (Fig. 1). Briefly, the *Legionella*-calibrated suspension was introduced into the nebulizer reservoir. The nebulizer was connected to the low-pressure impactor (DLPI) by an artificial throat. A pump-simulating inspiration was used during nebulization to collect aerosols on the 13-DLPI stages. The size classification in DLPI was set at up to 10 μm, with evenly distributed impactor stages.

**Figure 2:**
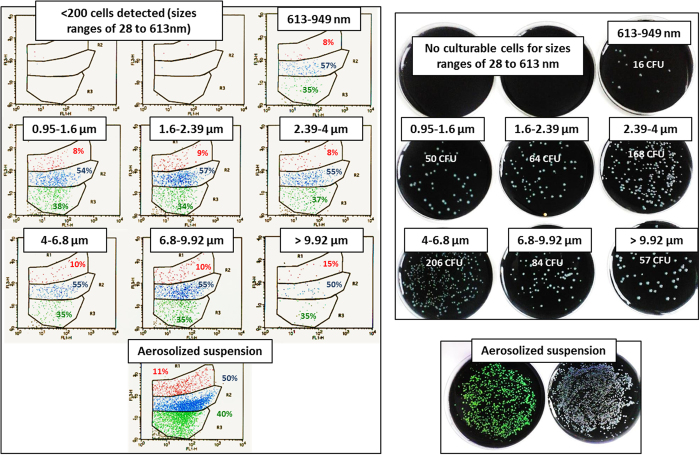
Representative experimental results obtained with FCA and culture for different aerodynamic size ranges of airborne particles (n = 3, repeated twice). Left: FCA results in percentages. Cells are distributed according to propidium iodide fluorescence intensity *versus* GFP fluorescence intensity. Green: VC, blue: VBNC, red: DC. Right: culture in colony-forming units (CFU). Aerosolized suspension of *Lp1 008* EPF cells at 2.10^7^ CFU/mL.

Bacterial detection and viability before and after aerosolization were assessed using culture, qPCR and FCA on *Lp1 008* suspensions of different concentrations and stages: exponential phase forms (EPFs) and stationary phase forms (SPFs) according to a procedure described by Faulkner and Garduno^27^. As determined by qPCR, only 15.8 ± 2.4% of the initial total inoculum (in genomic units (GU)) was recovered from the 13-DLPI stages after aerosolization. The remaining part of the inoculum was either kept in the nebulizer reservoir or disseminated in the artificial throat. The percentage recovered was the same for both suspensions at both concentrations, indicating good reproducibility of the nebulization process. As shown by FCA analysis and cultivability on BCYE media, the percentages of VC (35.7 ± 1.3%), VBNC (55.7 ± 1.1%) and dead cells (DC; 8.7 ± 0.9%) were the same for both aerosolized suspensions and those recovered at each DLPI stage (Fig. 2).

### Validation of bacterial suspension prior to aerosolization

**Figure 3:**
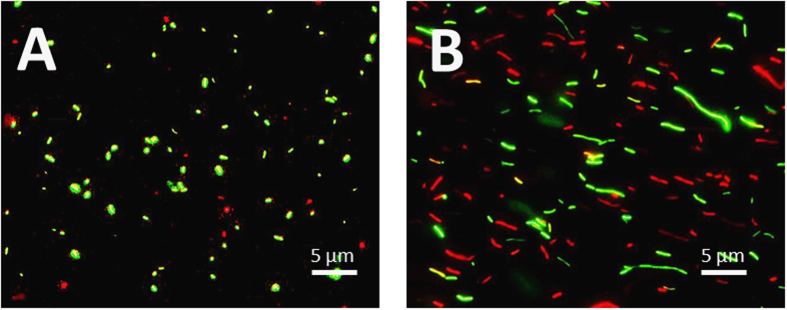
Microscopic epifluorescent image (Gx400) of aerosolized *Legionella* suspension. (**A**) *Lp1 008* EPF cells. (**B**) *Lp1 008* SPF cells.

Figure 3 shows that *Lp1 008* EPF cells were composed mostly of cells approximately 1 μm long whereas *Lp1 008* SPF cells consisted of polymorphic bacilli ranging from 1 μm to >5 μm. It is known that *Legionella* become highly polymorphic with age^27^. Because the size of the contaminating bacteria may be a critical factor with respect to both bioaerosol infection and access to the respiratory tract and small bacilli 1 μm long are often encountered within environmental samples as planktonic cells^28^,^29^, the use of *Lp1 008* EPF cell suspensions seemed more pertinent for further testing.

After FCA, more *Legionella* DC were observed in *Lp1 008* SPF cell than in *Lp1 008* EPF cell suspensions at the two concentrations tested (2 × 10^6^ and 2 × 10^7^/mL). Because the detection limit of FCA is 10^3^ cells/mL, the use of a calibrated suspension at 2 × 10^6^ CFU/mL was not appropriate. These results validated our choice to use *Lp1 008* EPF cell suspensions at 2 × 10^7^ CFU/mL for the characterization of *Legionella* aerosols.

### Size distribution of transporting droplets

**Figure 4:**
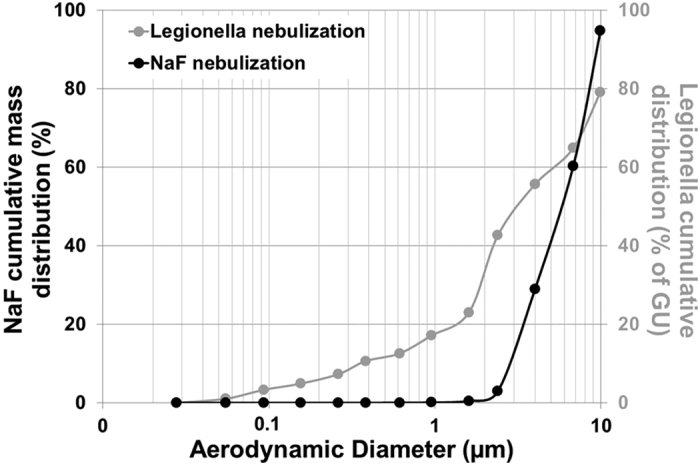
Typical cumulative distribution of airborne droplets characterized by a low-pressure impactor (DLPI). NaF quantification by an electrochemical method (expressed in % of mass reaching each stage of the DLPI, i.e., in % of total mass of NaF aerosolized). *Legionella* quantification by qPCR (expressed in % of genetic units reaching each stage of the DLPI, i.e., in % of total airborne *Legionella*). Particles larger than 10 μm were not plotted.

To determine whether sodium fluoride (NaF) and *Legionella* nebulization generate distinctive aerosols, they were compared with respect to cumulative distribution (NaF cumulative mass distribution *versus Legionella* cumulative distribution in % of genomic units). Figure 4 shows aerodynamic diameters, which, for unit density spheres such as water droplets, are the same as physical diameters.

The aerosols produced using NaF as a chemical marker for size distribution of transporting droplets demonstrated a mass median aerodynamic diameter (MMAD) of 6.1 μm ± 0.4 with a geometric standard deviation (GSD) of 1.7 μm ± 0.1. Thus, the size distribution of transporting droplets generated by the vibrating-mesh nebulizer can be considered to be monodispersed. In addition, the distribution of airborne *Legionella* showed an MMAD of 3.4 μm ± 0.4 with a GSD of 4.1 μm ± 1.2. The particle size distribution of *Legionella* generated by the nebulizer is highly polydispersed in comparison with the transporting droplets. This difference in the polydispersity index should be due to different compositions of the aerosols (e.g., bacteria packaged or not in droplets), as measured during nebulization using the vibrating mesh technology.

### Characterization of *Legionella* aerosols

Particles with aerodynamic diameters in the submicron range cannot transport *Legionella* bacteria, which are several times larger than the water-transporting particles. Moreover, a liquid environment appears to be necessary for the bacteria to remain viable while airborne. Thus, we assumed that the bacterial spread that may cause LD requires the generation of water droplets small enough to be deposited in the thoracic region but large enough to contain viable and virulent *Legionella*. The size distribution of airborne droplets is critical for delivering viable bacteria to the respiratory tract, and the mass of droplets of this given size is related to the number of bacteria contained within. A specific size range of droplets (2–10 μm) is required for the bacteria to be delivered to the thoracic region^30^. Overall, one can admit that the minimum droplet diameter that may contain bacteria is between 1 and 2 μm^14^,^15^. However, depending on the way by which aerosols are inhaled (mouth *versus* nose inhalation of aerosols), results may vary. Indeed, several studies have shown that nose-inhalation aerosols of more than 2.5 μm in diameter cannot reach the pulmonary alveoli^31^,^32^. Mouth inhalation of aerosols may propose different results, showing the possibility for aerosols of 1–10 μm to reach the pulmonary tract^33^.

In the present study, 93.4% of the total amount of airborne *Legionella* DNA was in a particle size range of 1.6–9.92 μm (Fig. 4, Tables 1 and 2). This result indicates that 93.4% of the airborne *Legionella* DNA detected were compatible with efficient aerosolization of bacteria entrapped in water-transporting particles. The particles were large enough to contain *Legionella* in viable and pathogenic conditions and to allow droplet deposition in the thoracic region.

We suggest two possible explanations why the particle size of the remaining 6.6% (1.1%, 1.7% and 3.8% in the 28–262 nm, 262–949 nm and 0.949–1.6 μm ranges, respectively) of the total airborne *Legionella* DNA was 55 nm-1.6 μm (Fig. 4, Table 1). One is that the *Legionella* DNA detected might come from cellular fragments that are entrapped or not in droplets. This phenomenon can occur for all particle sizes, but it seems uncommon; approximately 1.1% of the total airborne *Legionella* DNA could be attributed to cellular fragments of 28–262 nm because no culturable cells were detected (Fig. 2, Tables 1 and 2). The second possible explanation is that the orientation of bacteria against the flow direction during sampling in the DLPI cascade impactor resulted in the presence of 1.7% of total airborne *Legionella* DNA, with the detection of culturable cells of 0.262–0.949 μm (Fig. 2, Tables 1 and 2). This seems to indicate that some airborne and viable *Legionella* cells were not packaged in spherical liquid droplets (because it is sterically impossible to entrap a viable *Legionella* cell in a sphere 0.3 μm in diameter). Indeed, the gravitational settling speed of a particle is determined by the opposing effects of the gravitational force and the aerodynamic resistance. The aerodynamic drag force depends on the shape and orientation of the particle with respect to its direction of motion. Thus, a cylindrical particle settles under gravity with an orientation parallel to its direction of motion. This phenomenon was observed with inhalation of fibrous particles such as asbestos^34^.

The detection of culturable cells from the 262 nm cut-off diameter of the DLPI cascade impactor clearly shows that viable bacteria that were not entrapped in droplets were aerosolized in the 262–949 nm range. Specifically, the detection of culturable cells of 262 nm is to be expected if we consider the orientation of the bacteria with respect to their direction of motion in the cascade impactor, given the bacteria’s rod-like shape and their size (approximately 1–2 μm long and 0.3–0.9 μm wide). Finally, the 3.8% of the total airborne *Legionella* DNA that were detected in the size range of 0.949–1.6 μm can be attributed to airborne *Legionella* in transporting droplets (entrapped or not) because the droplet diameter that may contain these bacteria is between 1 and 2 μm^14^,^15^.

Thus, we can identify 4 modes of transportation during the aerosolization of *Legionella* (Table 2): cellular fragments of *Legionella* (entrapped or not) in droplets (28–262 nm), airborne bacteria not entrapped in droplets and oriented by the air flow direction during the respiration process (0.262–0.949 μm), airborne bacteria (entrapped or not) in droplets (0.949–1.6 μm), and airborne bacteria packaged in droplets (1.6–9.92 μm).

In our study, 44% of total airborne *Legionella* DNA or 7% of initial bacterial suspension in the 0.26–2.5 μm range reached pulmonary alveoli. Among viable forms (VC and VBNC) as determined by FCA and cultivability, 43% of total airborne viable *Legionella* or 0.7% of initial viable bacterial suspension reached the pulmonary alveoli (Tables 1 and 2). As shown in microscopic epifluorescent images (Gx400) of aerosolized *Legionella* suspensions (Fig. 3), *Lp1 008* EPF cells were composed mostly of cells approximately 1 μm long, whereas *Lp1 008* SPF cells consisted of polymorphic bacilli ranging from 1 μm to >5 μm in size. Consequently, only *Lp1 008* EPF cells could reach the pulmonary alveoli.

Few attempts have been made to investigate the sizes of liquid aerosols that have disease potential. Insights into the human dose response for LD are determined by quantitative microbial risk assessment (QMRA) techniques that animal model data may provide^35^. However, injected *Legionella* concentrations were determined as culturable cells (VC) only^36^,^37^,^38^, although real concentrations of total viable cells (VC and VBNC) can be determined by FCA^23^. Under our experimental conditions, 0.7% of initial viable bacterial suspensions might reach the pulmonary alveoli. Thus, it is possible to standardize the size of inoculum injected in animal models by reducing the high dispersion of the LD50% (lethal dose) values^35^ and focusing on all viable forms (VC and VBNC).

Animals are infected via intratracheal, nasal, intraperitoneal injections or tracheal instillation. This study is the first investigation of the aerosolization of *Legionella* by vibrating-mesh technology, which enables delivery of *Legionella* aerosol to a laboratory animal model.

## Methods

### Experimental design

Results presented in the tables and figures are for a bacterial concentration of 2.10^7^ CFU/mL (2–3 days with *Lp1 008* EPF cells). The aerosol experiments were performed in triplicate (3*13-DLPI stages, i.e., 39 analyzed samples). The biological analysis of each 13-DLPI stage was performed in duplicate (i.e., 78 samples for culture, FCA and qPCR).

### Bacterial culture and suspension preparation

A *Legionella pneumophila* serogroup 1 GFP (green fluorescent protein)-modified strain was used^39^,^40^. This *LP1 008*-GFP strain was stored at −80 °C in Cryobank tubes (Mast Diagnostic, Amiens, France). After thawing, the strain was plated onto BCYE agar (Buffered Charcoal Yeast Extract, Oxoid, France) supplemented with chloramphenicol (Sigma Aldrich, France) at 8 mg/mL for 72 h at 37 °C (for plasmid selection) and then re-plated onto the same medium and incubated at 37 °C for another 2–3 days to obtain *Lp1 008* EPF cells or for 5–6 days to obtain *Lp1 008* SPF cells. These cultures were then used to achieve 5 mL suspensions in sterile normal saline (0.9% NaCl) water at a final concentration of 2.10^6^ or 2.10^7^ CFU/mL. An optical density of 0.2 at 600 nm (Biomate TM3; Avantec, Illkirch, France) was taken as a reference for a suspension containing 10^8^ CFU/mL. The 5 mL suspensions were immediately placed in the nebulizer for cascade impactor experiments to determine the size distribution of airborne particles.

### Generation of aerosols

Nebulizers for medical applications are usually employed to generate bioaerosols with small-particles (1–3 μm)^14^. In this study, the nebulizer used to aerosolize bacterial suspensions, or sodium fluoride solution (used as a usual chemical tracer for aerosol according to the NF EN 13544-1 standard), was an e-Base^®^ Nebulizer System (PARI GmbH, Stanberg, Germany). The mechanism of aerosol generation was based on a perforated oscillating membrane technology. It consists of the high-frequency vibration of a very thin membrane with a multitude of micrometric holes. The holes allow very fine nebulization from the liquid in the device’s reservoir (Fig. 1).

### Size distribution of airborne particles

#### Low-pressure cascade impactor set-up

The airborne particle size distribution was assessed using a 13-stage cascade low-pressure impactor (DLPI, Dekati, Finland). At an airflow of 10 L min^−1^, particles were impacted depending on their inertia-related aerodynamic diameter in one of the 13 size fraction stages. The size classification in DLPI was set at up to 10 μm, with evenly distributed impactor stages. In each size fraction, the particles were collected on collection substrates to obtain their gravimetric size distribution. Specifically, the mass median aerodynamic diameter (MMAD) and the geometric standard deviation (GSD) were calculated. The MMAD was defined as the median of the distribution of the airborne particle mass with respect to the aerodynamic diameter. The GSD was used to characterize the dispersion of aerosols. When the GSD was lower than 2, aerosols were considered to be monodispersed; when it was higher than 2, the particle size distribution was polydispersed^32^,^41^,^42^.

#### Size distribution of transporting droplets

We used NaF as a molecular tracer for aerosols in order to determine the aerodynamic size distribution of the droplets according to the NF EN 13544-1 standard. For this purpose, 4 mL of NaF (2.5 wt%) was introduced into the nebulizer reservoir. The nebulizer was connected to the DLPI via an artificial throat (according to the United States Pharmacopeia: height 112 mm, width 42 mm, internal diameter 19 mm) (Fig. 1). The NaF solution initially introduced in the reservoir was aerosolized for 10 min. Then, each DLPI stage was rinsed with 5 mL of deionized water to collect the NaF deposited on each size-fraction stage. The NaF concentration of samples was assayed using an electrochemical method (PerfectIONTM combined with a SevenGo proTM F− electrode, Mettler Toledo, France). Finally, the MMAD and the GSD of the nebulized droplets were calculated^32^,^41^,^42^.

#### Size distribution of airborne-transported bacteria

A bacterial analysis of the size-classified particles was performed when a bacterial suspension was introduced into the nebulizer reservoir. Indeed, transporting water droplets must be large enough to contain *Legionella* in viable conditions. Thus, the concentration of *Legionella* bacteria depending on the water droplet inertia-related aerodynamic diameter should also be determined. These experiments were conducted inside a Glove Box (815-PGB GLOVE BOX, Fisher Scientific) to prevent infection of the experimenters. The same DLPI protocol as that used for the NaF aerosol experiments was employed, except that each stage of the cascade impactor was covered with a specific membrane for *Legionella* collection (polycarbonate membrane–membrane filters Nuclepore™ track etched, Whatman, GE Healthcare). After nebulization, each membrane potentially retaining bacteria was collected in 5 mL of physiological water. After 30 sec of sonication (Bransonic 32 sonicator bath, Branson Instruments, Danbury, CT USA), the bacterial suspension of each 13-DLPI stage was analyzed by culture (viable and culturable cells—VC), flow cytometry (percentage of VBNC cells) and qPCR (VC, VBNC and dead cells—DC).

### Biological characterization of *Legionella* bacteria

#### Quantification by qPCR

The bacteria were quantified using a GFP mut2 sequence^43^ expressed by *Lp1 008*-GFP cells. The forward and reverse primers were 5′-AGAGTGCCATGCCCGAAGGT-3′ and 5′-AAGGACAGGGCCATCGCCAA-3′, respectively. Plasmid DNA was extracted from all samples with the NuCleoSpin Plasmid kit (Macherey-Nagel) according to a procedure described by Cormack *et al*. Real-time quantitative PCR (qPCR) was carried out on an ABI Prism 7500 automate (Applied Biosystems), using the 2X Power SYBR^®^ Green PCR Master Mix (Life Technologies) under the following conditions: initial denaturation for 15 min at 95 °C, 50 cycles consisting of 15 s denaturation at 95 °C, and 1 min annealing and elongation at 60 °C. At the end of every elongation step, the fluorescence of the incorporated SYBR green dye was measured. After 50 cycles of amplification, a melting curve program was incorporated to check for any primer dimers or other non-specific amplifications. For each experiment, three independent extractions, on the initial calibrated suspension and on each 13-DLPI stage, were performed, and each extraction was run in duplicate. The results were analyzed using Sequence Detection Software version 1.4 (ABI 7500 System Software, Applied Biosystems). The results were expressed in genomic units (GU) of amplified GFP sequence.

#### Cultivability

Cultivability assessment (determination of the number of viable and culturable cells—VC) was performed on initial bacterial suspensions and on bacterial suspensions collected from each 13-DLPI stage. Experiments were performed in duplicate. Each bacterial suspension (100 μL) was plated on two BCYE agar samples with or without chloramphenicol supplementation and incubated at 37 °C for 72 h. All BCYE plates were observed under UV at 366 nm to enumerate *Legionella* colonies (CFU) expressing GFP.

#### Percentage of VBNC Legionella by flow cytometric assay (FCA)

As previously described^20^,^23^,^24^, FCA profiles were obtained using a combination of GFP green fluorescence (viable cells expressing GFP) and propidium iodide (PI) red fluorescence for cells with damaged membranes. One milliliter of bacterial suspension (prior aerosolization or collected from each 13-DLPI stages) was labeled with 5 μL PI at 1 mg/mL. Flow cytometric measurements were performed using a BD FACSCalibur instrument (Becton Dickinson Biosciences) equipped with an air-cooled argon laser (488 nm emission; 20 mW). The green fluorescent emission from GFP was collected in the FL1 channel (500 to 560 nm), and the red fluorescence from PI was collected in the FL3 channel (670 nm). A threshold was applied to the FL1 channel to eliminate background signals. Analysis was performed at a low-flow-rate setting. The results were analyzed using Cell Quest Pro software (Becton Dickinson Biosciences).

## Additional Information

**How to cite this article**: Allegra, S. *et al*. Characterization of aerosols containing *Legionella* generated upon nebulization. *Sci. Rep*. **6**, 33998; doi: 10.1038/srep33998 (2016).

## Figures and Tables

**Table 1:** Quantification of *Legionella* by qPCR (expressed as percentages of genomic units) reaching each stage of the DLPI.

Range of aerodynamic diameter	*Legionella* in % of the initial bacterial suspension	*Legionella* in % of the total airborne bacteria
28–55 nm	**0.01 ± 0.02**	**0.10 ± 0.80**
55–93 nm	**0.04 ± 0.05**	**0.25 ± 1.30**
93–155 nm	**0.11 ± 0.05**	**0.65 ± 0.70**
155-262 nm	**0.02 ± 0.05**	**0.10 ± 1.50**
262–382 nm	0.07 ± 0.05*	0.45 ± 1.60*
382–613 nm	0.11 ± 0.15*	0.70 ± 2.70*
613–949 nm	0.09 ± 0.10*	0.55 ± 2.20*
949–1600 nm	0.60 ± 0.20*	3.80 ± 5.40*
1.6–2.39 μm	5.85 ± 2.20*	37.15 ± 8.20*
2.39–4 μm	1.20 ± 0.40*	7.80 ± 1.85*
4–6.8 μm	0.85 ± 0.70*	5.40 ± 7.55*
6.8–9.92 μm	2.40 ± 0.90*	15.15 ± 5.40*
>9.92 μm	4.40 ± 1.70*	27.90 ± 9.15*

Total (VC, VBNC and DC) *Lp1 008 Legionella* was quantified by qPCR. The results from three independent experiments are expressed as percentages of genomic units (GU) compared to the quantity of total *Legionella* in bacterial suspension initially introduced into the nebulizer to generate the bioaerosol or compared to the total aerosolized *Legionella* (i.e., the sum of the bacteria deposited in the 13 stages of the DLPI). Sizes of aerosols that may reach the pulmonary region are highlighted in bold.

^*^Culturable cells.

**Table 2:** Main features of *Legionella* bioaerosols depending on the transporting droplet size distribution.

		0–0.262	0.262–0.949	0.949–1.6	1.6–9.92
Droplet range (μm)			Possibility of cellular fragments entrapped or not in droplets		
Mode of bacterial transportation		Cellular fragments (entrapped or not) in droplets	Airborne bacteria not entrapped in droplets	Airborne bacteria (entrapped or not) in droplets	Airborne bacteria packaged in droplets
Total *Legionella* quantification by qPCR	in % of the initial bacterial suspension	0.2 ± 0.1	0.3 ± 0.1	0.6 ± 0.3	14.7 ± 2.1
	in % of the total airborne bacteria	1.1 ± 0.3	1.7 ± 0.1	3.8 ± 0.4	93.4 ± 14.2
Detection of culturable cells		No	Yes	Yes	Yes
Viable cells in CFU		Not detectable	2 × 10^4^ ± 1.4 × 10^3^	4.5 × 10^4^ ± 1.8 × 10^4^	1.1 × 10^6^ ± 1.6 × 10^5^

Total *Legionella* (VC, VBNC, DC) results are expressed as percentages (see Table 1). Viable cells (VC, VBNC) results from three independent experiments are expressed in colony-forming Units (CFU).
